# Proceedings of the 2017 MidSouth Computational Biology and Bioinformatics Society (MCBIOS) Conference

**DOI:** 10.1186/s12859-017-1887-2

**Published:** 2017-12-28

**Authors:** Jonathan D. Wren, Mikhail G. Dozmorov, Inimary Toby, Bindu Nanduri, Ramin Homayouni, Prashanti Manda, Shraddha Thakkar

**Affiliations:** 1 Arthritis and Clinical Immunology Research Program, Division of Genomics and Data Sciences, Oklahoma Medical Research Foundation, 825 N.E. 13th Street, Oklahoma City, OK 73104-5005 USA; 2 Biochemistry and Molecular Biology Dept, Virginia Commonwealth University, 830 East Main Street, Richmond, Virginia, 23298 USA; 3 Stephenson Cancer Center, Virginia Commonwealth University, 830 East Main Street, Richmond, Virginia, 23298 USA; 4 Department of Geriatric Medicine, University of Oklahoma Health Sciences Center, Virginia Commonwealth University, 830 East Main Street, Richmond, Virginia, 23298 USA; 5 Department of Biostatistics, Virginia Commonwealth University, 830 East Main Street, Richmond, Virginia, 23298 USA; 6 Department of Clinical Sciences, UT Southwestern Medical Center, 5323 Harry Hines Boulevard, Dallas, TX 75390-9066 USA; 7 Department of Basic Sciences, College of Veterinary Medicine, Mississippi State University, Mississippi State, MS 39762 USA; 8 Department of Biological Sciences, Bioinformatics Program, University of Memphis, Memphis, TN 38152 USA; 9 Department of Computer Science, University of North Carolina at Greensboro, Greensboro, NC USA; 10 Division of Bioinformatics and Biostatistics, National Center for Toxicological Research, US Food and Drug Administration, Jefferson, AR 72079 USA

**Keywords:** Bioinformatics, Conferences, MCBIOS, ISCB

## Introduction

The XIVth Annual MidSouth Computational Biology and Bioinformatics Society was held in Little Rock, AR From March 23-25st 2017 and was co-hosted by University of Arkansas at Little Rock, University of Arkansas for Medical Sciences, Little Rock, AR and National Center for Toxicological Research, Jefferson, AR. The fourteenth annual conference entitled “*Make them Safer Make them Better: Bioinformatics and the Development of Therapeuticals*”. There were 220 conference registrants and 129 abstracts submitted, including 62 oral and 67 poster presentations.

The conference was co-chaired by Cesar M. Compadre, Ph.D. from UAMS and William Slikker Jr., Ph.D. from FDA/NCTR. The program was co-chaired by Shraddha Thakkar, Ph.D., and Weida Tong, Ph.D., from Division of Bioinformatics and Biostatics of FDA/NCTR Conference committee members were Darin E. Jones, Ph.D., Assistant Professor and Mary Yang, Ph.D., from UALR, Miss Ujwani Nukala, MS, from university of Arkansas at Little Rock, AR. For 2018–9, Dr. Ramin Homayouni, Ph.D. from University of Memphis, Memphis, TN was chosen as President-Elect and Bindu Nanduri, Ph.D., from Mississippi State University, Starkville, MS as President.

**Keynote speakers were:**

**Day 1: March 23rd 2017**

“Pharmacogenetic and Genomic applications for Safety and Therapeutic Efficacy Assessment in drug development programs” by Prof. Dr. Jürgen Borlak, Hannover Medical School, Hannover, Germany.

**Day 2: March 24th 2017**

“The Top 5 Greatest Bioinformatics Graphs Never Published” by Wendell Jones, Ph.D., Q2 Solutions | EA Genomics, Morrisville, North Carolina.

**Day 3: March 25th 2017**

“Targeting Undruggable Protein Tyrosine Phosphatases” by John Lazo, Ph.D., Professor in Pharmacology, University of Virginia, Charlottesville, VA.

**The conference program included four workshops:**

**Workshop 1:** MedDRA, by Anna Zhao-Wong, Ph.D.

**Workshop 2:** Next-Generation sequencing using Galaxy, by Binsheng Gong, Ph.D.

**Workshop 3:** PubChem, by Yanli Wang, Ph.D.

**Workshop 4:** Next-Generation Sequencing and Bioinformatics by Wenming Xiao, Ph.D.

**There were 9 breakout sessions. Topics and facilitators were:**

**Breakout Session I:** Metagenomics and the Microbiome, Carl E. Cerniglia, Ph.D.

**Breakout Session II:** Biomedical Informatics, Fred Prior, Ph.D.

**Breakout Session III:** Machine Learning and Chemoinformatics, Joshua Swamidass, MD, Ph.D.

**Breakout Session IV:** Drug Design and Development, Cesar Compadre, Ph.D.

**Breakout Session V:** Biomarker and high-throughput data analysis, Mary Yang, Ph.D.

**Breakout Session VI:** Genomics and therapeutics development, Zhichao Liu, Ph.D.

**Breakout Session VII:** Reproducible Genomics and Toxicogenomics, Joshua Xu, Ph.D.

**Breakout Session VIII:** in silico and in vivo Adverse Reaction Detection, Minjun Chen, Ph.D.

**Breakout Session IX:** Systems pharmacology and Bioinformatics, Jake Chen, Ph.D.

**Best Paper Award, MCBIOS 2017**: Cory Giles et al. “ALE: Automated Label Extraction from GEO metadata” [1].

This year MCBIOS launched the “MCBIOS Young Scientist Excellence” awards to recognize students and postdoctoral fellows that exhibit scientific excellence in the field of Bioinformatics. Student and postdoctoral fellows to go through a rigorous award application and the top candidates give a plenary presentation on the first day of the conference. To be able to compete, students submitted an abstract with separate descriptions of the innovations in the research and their specific roles in carrying out the work. Candidates were first evaluated by the MCBIOS board members and then by a panel of judges (including keynote speakers), who evaluated applications for the quality and impact of the research. The quality of professional presentation is the primary consideration for receiving the award as well as creativity, dedication, and multidisciplinary contributions demonstrated by the candidates. The idea was to select candidates with demonstrated multidisciplinary contributions and initiative.

**Post-Doctoral winners of the MCBIOS Young Scientist Excellence award**

**First Place (tie):**

Harsh Dweep, Ph.D.

Institution: US FDA/NCTR.

Title of the presentation: Defining a Landscape of Genes, miRNA and their Associations in Hepatocarcinogenesis: A Study of Thioacetamide with Multiple Doses and Time Intervals.

Tanmay Bera, Ph.D.

Institution: US FDA/NCTR.

Title: Developing Image Analysis Methods to Identify the Species of Food Contaminating Beetles.

**Second Place:**

Name: Suxing Liu, Ph.D.

Institution: Arkansas State University.

Title: Novel Low Cost 3D Surface Model Reconstruction System for Plant Phenotyping.

**Third Place:**

Name: Suguna Devi Sakkiah.

Institution: US FDA/NCTR.

Title: Development and Validation of Estrogen Receptor Beta Binding Prediction Model Using Large Sets of Chemicals.

**Student winners of the MCBIOS Young Scientist Excellence award**

**First Place:**

Name: Brian Delavan.

University: University of Arkansas of Little Rock (UALR) and US FDA/NCTR.

Title: Drug Repurposing for LEOPARD Syndrome by Integrating Chemical Structure and Genomics based Approaches.

**Second Place:**

Name: Ujwani Nukala.

University: UALR and UAMS.

Title: Development of novel vitamin E analogs, Tocoflexols with enhanced bioavailability using in silico approaches.

**Third Place:**

Name: Shahin Boluki.

University: Texas A&M University.

Title: Prior Construction for Optimal Bayesian Classification Using Unlabeled Data.

**Poster session award:**

The poster session was held at the end of the first day of the meeting. Student and post-doctoral presenters presented their work at the poster sessions and it was judged for presentation quality by a panel of MCBIOS professional members that attended the conference.

**Student Winners:**

**First Place:**

Name: Priyam Patel

University: University of Memphis.

Title: Automated Bioinformatics Analysis Package using System On Chip (ABAPSoC).

**Second Place (tie):**

Name: Alan Amaya

University: East Tennessee State University.

Title: Analyzing the Protein-Protein Interaction Network of Tnf-Alpha.

Name: Bryan Naidenov

University: Oklahoma State University.

Title: Novel Gene Discovery by Genome Completion through De Novo Assembly of Long-Reads.

**Third Place**

Name: Anqi Walbaum.

University: UAMS.

Title: Computational Modeling of the Interaction of Novel Quaternary Ammonium Molecules with the α9β10 Nicotinic Acetylcholine Receptor.

**Post-Doctoral Winners for the poster presentation**

**First Place**

Name: Patrick Apopa, Ph.D.

University: UAMS.

Title: The Role of Fastkd3 Gene Expression In Oncogenesis of Non-Small Cell Lung Carcinoma.

**Second Place**

Name: Shuzhen Sun, Ph.D.

University: Oklahoma State University.

Title: SNP Variable Selection by Generalized Graph Domination.

**Third Place (tie)**

Name: Thidathip Wongsurawat, Ph.D.

University: UAMS.

Title: R-loop Forming Structure Prediction in Viral Genomes.

Name: Dianke Yu

Institution: FDA/NCTR.

Title: Roles of Long Non-Coding RNAs In Acetaminophen-Induced Liver Injury.

## Selecting papers for the MCBIOS XI proceedings

From the work presented at MCBIOS 2017, a total of 24 papers were submitted to be considered for publication in this year’s Proceedings, and 15 papers were accepted (63% acceptance rate). At least 2 reviewers anonymously peer-reviewed all submitted papers and acceptable papers were quantitatively ranked on the basis of three evaluation criteria: Novelty (1–5), Impact (1–5) and Clarity (1–3). Editors that were co-authors of submitted papers were not permitted to handle their own papers editorially. Papers generally fell into three categories:

## Networks and microbial communities

Zongliang Yue et al. applied a systems pharmacology framework to reposition drugs for polygenic diseases, in this case Parkinson’s disease (PD) [2]. Integration of GWAS and gene expression data in the context of regulatory networks enabled the identification of PD-specific modules that can be targeted through drug repositioning with a score based on known drug-gene activity profiles.

Hyundoo Jeong et al. report CUFID-query, software for local network alignment, whereby one can query network modules against larger networks [3]. CUFID-query will detect conserved functional modules within a large network that are expected to perform similar functions to the queried subnetwork on the basis of how nodes are interconnected.

Quang Minh Tran et al. address a challenging problem in metagenomics by identification and quantification of microbial genomes from unknown bacteria from environmental samples using next-generation sequencing information [4]. For this analysis they used 16S rRNA instead of whole genome. They demonstrated in the manuscript that, accurate and robust predictions can be made at different read coverage and percentage of unknown bacteria.

## Genomics & Transcriptomics

Li and Yang report on an approach to identify orthologous long non-coding RNAs (lncRNAs) [5]. Unlike proteins, lncRNAs tend to have much less sequence conservation, and are harder to identify. Focusing on lncRNAs in human vs rat brain tissue, they identify 140 new lncRNAs not present in the existing databases.

Keqin Liu et al. investigated the role of non-significantly mutated cancer genes that remain underexplored due to hard significance thresholds [6]. The study demonstrates that non-significantly mutated genes in endometrial cancer can effectively classify histological subtypes, predict clinical outcomes, and are enriched in relevant signaling pathways. The results suggest that less significant gene mutations should be considered along with the more significant ones.

Se-ran Jun et al. present an in-silico study of the Zika Virus genome, focusing specifically on the contrast between the Brazilian strain, unique for causing microcephaly, and the Asian and African strains [7]. They use a robust set of Zika viral genome data and compare their findings to those of other groups that have worked with different subsets of this data and address the occasional divergence in the results/conclusions that currently exist in the Zika literature.

Chun-Chi Chen et al. develop a novel method for predicting piRNAs in genome sequences [8]. The method classifies piRNAs based on shared sequence motifs and identifies predictive features using n-gram models. They demonstrate the algorithm using evaluations in three species – *Homo sapiens*, *Rattus norvegicus*, and *Mus musculus*.

Ethan Rath et al. aimed to identify trans-acting sRNAs that can be substrates of RNaseIII by comparing the RNase III gene deleted mutants with the Streptococcus pyogenes wild-type using RNA-seq data [9]. To achieve that, they developed a custom script that can detect reads that support the intergenic regions of the S. pyogenes genome. With their analysis they were able to identify the novel sRNAs to expand understanding of the regulatory elements involved in S. Pyogenes.

Cory Giles et al. developed a tool for automated extraction of labels such as gender, tissue, etc. for GEO data [1]. They also present a tool for predicting missing labels using probabilistic measures via gene expression data. They find that first assigning labels using heuristic text-extraction approaches enables the creation of larger training datasets for downstream machine learning models, and achieves better label prediction.

## Machine learning

The manuscript by Recep Erol et al. reports an improved computational approach to detect malignancy in skin lesions from dermatoscopic images [10]. Their method utilized Level Set Propagation (LSP) to detect abrupt lesion boundaries. The texture features of the lesions were then used in several different machine learning classifiers and evaluated for accuracy of malignancy detection. Using a fully-connected multi-hidden layer Neural Net classifier they achieved a specificity of 78%.

Maxwell et al. compared Deep learning Neural Networks (DNN) with standard multi-label classification methods for classifying chronic diseases such as diabetes, hypertension and fatty liver from anonymous medical records from over 110,300 for intelligent health risk prediction. DNNS had the highest accuracy compared to SVM and MLKNN classifiers [11].

Shahin Boluki et al. introduce a new method called Maximal Knowledge-Driven Information Prior (MKDIP), which utilizes an Optimal Bayesian Classifier framework to integrate gene regulatory and pathway knowledge for phenotypic classification [12]. The performance of MKDIP was favorable compared to several Bayesian and non-Bayesian classification methods using two well-known pathways and a gene expression dataset on non-small cell lung cancer.

Mohsen Sharifi et al. develop a machine-learning method to predict which drugs could cause a potentially life-threatening arrhythmia known as Torsade de Pointes (TdP) [13]. The method uses 3-dimensional spectral data-activity relationships (3D–SDAR) to identify molecular features responsible for the structure-activity relationship between drugs and the hERG receptor. The contribution of this method is to enable new drugs to be screened early against their potential to cause cardiac arrhythmias, which is a major cause for eventual failure of new drugs.

## Miscellaneous

Visanu Wanchai et al. present a web-friendly interface that provides all available bacterial organisms from major public databases taken from several annotation sources including draft genomes [14]. The tool offers users analysis and visualization capabilities whereby quality scores can be utilized as metrics for downstream assessments of bacterial genome comparisons. Quality scores are calculated using the following methods: assembly quality, number of rRNA, and tRNA genes and the occurrence of conserved functional domains.

Mikailov et al. report on a new parallelization method to remove limitations of multi-threading and Message Passing Interface parallelization techniques, scale bioinformatics applications performing sequence search and alignment across a HPC cluster, and adds checkpointing capabilities. This method, referred to as a “dual segmentation” method, is based on segmentation of both query and reference database combining partial solutions published earlier. Applying this method, BLAST run time fell from 27 days to <4 h [15].

## Future meetings

The 15th Annual MCBIOS conference will be held in Starkville, Mississippi from March 29th-31st, 2018.
